# Incidence and Evaluation of Incidental Abnormal Bone Marrow Signal on Magnetic Resonance Imaging

**DOI:** 10.1155/2014/380814

**Published:** 2014-10-14

**Authors:** Gunjan L. Shah, Aaron S. Rosenberg, Jamie Jarboe, Andreas Klein, Furha Cossor

**Affiliations:** ^1^Adult Bone Marrow Transplantation Service, Department of Medicine, Memorial Sloan Kettering Cancer Center, 1275 York Avenue, P.O. Box 298, New York, NY 10065, USA; ^2^Division of Hematology/Oncology, UC Davis Comprehensive Cancer Center, 4501 X Street, Suite 3016, Sacramento, CA 95817, USA; ^3^Graves Gilbert Clinic, 201 Park Street, Bowling Green, KY 42102, USA; ^4^Tufts Medical Center, 800 Washington Street, P.O. Box 245, Boston, MA 02111, USA; ^5^Lahey Hospital & Medical Center, 41 Mall Road, Burlington, MA 01805, USA

## Abstract

*Purpose*. The increased use of magnetic resonance imaging (MRI) has resulted in reports of incidental abnormal bone marrow (BM) signal. Our goal was to determine the evaluation of an incidental abnormal BM signal on MRI and the prevalence of a subsequent oncologic diagnosis. *Methods*. We conducted a retrospective cohort study of patients over age 18 undergoing MRI between May 2005 and October 2010 at Tufts Medical Center (TMC) with follow-up through November 2013. The electronic medical record was queried to determine imaging site, reason for scan, evaluation following radiology report, and final diagnosis. *Results*. 49,678 MRIs were done with 110 patients meeting inclusion criteria. Twenty two percent underwent some evaluation, most commonly a complete blood count, serum protein electrophoresis, or bone scan. With median follow-up of 41 months, 6% of patients were diagnosed with malignancies including multiple myeloma, non-Hodgkins lymphoma, metastatic non-small cell lung cancer, and metastatic adenocarcinoma. One patient who had not undergone evaluation developed breast cancer 24 months after the MRI. *Conclusions*. Incidentally noted abnormal or heterogeneous bone marrow signal on MRI was not inconsequential and should prompt further evaluation.

## 1. Introduction

Red to yellow bone marrow conversion occurs predictably with aging, with fatty replacement of cellular hematopoietically active marrow starting in the peripheral appendicular skeleton and proceeding to the central axial skeleton [1–3]. Reconversion to hematopoietically active marrow can occur in the setting of malignancy, necrosis, fibrosis, edema secondary to trauma or stress, replacement, infiltration, or infection, but the patient may be asymptomatic clinically [3, 4]. In addition, heavy smoking history has been significantly associated with marrow reconversion [5]. While reconversion can be seen noninvasively on MRI, the exact etiology is difficult to determine from imaging alone.

Over the past two decades, the use of magnetic resonance imaging (MRI) has increased annually [6], resulting in increased numbers of incidental findings [7–9]. The few studies that have examined the etiology of these findings found primarily benign lesions and rarely occult systemic malignancy [10–13]. Abnormal bone marrow (BM) signal is one such incidental finding and often prompts consult to hematology/oncology for evaluation of primary BM disorders. The prevalence of malignant diagnoses in such cases is unknown.

The purpose of our retrospective study of adult patients evaluated with MRI at Tufts Medical Center (TMC) was to determine the prevalence of a subsequent oncologic diagnosis and the appropriate evaluation for such findings as there is no standard in the published literature.

## 2. Methods

Between May 9, 2005 and October 31, 2010, the TMC Department of Radiology picture archiving and communication system (PACS) reports were searchable using GE Centricity Radiology Information System 2.2. Potential cases were identified by searching for MRI reports containing the phrases “abnormal bone marrow” or “heterogeneous bone marrow” during this time frame in patients over 18 years of age.

Cases in which the radiologist commented that there was no abnormal bone marrow signal or in which the patient had an underlying malignant diagnosis were excluded. Cases that remained for analysis either had no clear etiology of the abnormal signal stated in the report or clinical correlation was suggested by the radiologist. Patients undergoing MRIs of multiple anatomic sites on the same day prompting duplicate reports were included only once.

This study was performed with approval from the TMC Institutional Review Board and analyzed using Excel 2010 (Microsoft, Redmond, WA).

Demographics were collected from the electronic medical record (EMR). In addition, the ordering specialty, the anatomic location of the MRI, and the reason for the scan were abstracted from the radiology report. Reasons for the scan were combined into related categories. Musculoskeletal complaints included fractures and pain, including neck, shoulder, elbow, back, hip, knee, leg, or foot pain. Neurologic complaints included headaches, cerebrovascular accidents, transient ischemic attacks, multiple sclerosis, middle cerebral artery aneurysms, arm numbness and weakness, neurogenic claudication, leg weakness, radiculopathy, myelopathy, spinal stenosis, and paresthesias. Osteomyelitis, abscesses, and tuberculosis were categorized as infection. Prior abnormal imaging included scans done after findings on computed tomography scans and X-rays. Another category was created for the remainder of reasons, including axilla pain, pelvic pain, osteoporosis, hematuria, fibroids, evaluations for suspected pheochromocytomas and prolactinomas, soft tissue masses, and fibroids.

Further evaluation was defined as any referral to a subspecialist, laboratory test, imaging study, or procedure mentioned as part of an evaluation for this finding in an outpatient clinic note or inpatient discharge summary included in the TMC EMR after the incident MRI. Final diagnosis was defined by a pathology report confirming the diagnosis or a physician documenting the etiology of the abnormal signal. Time to hematology/oncology referral and time to final diagnosis were calculated from the date of the MRI. If no work-up was done or no diagnosis was determined in those patients undergoing evaluation, the medical record was examined for later diagnosis of a malignancy. Last follow-up was documented as of November 15, 2013.

Smoking status and hemoglobin less than 12 mg/dL documented within three months of the MRI were also collected to account for other differential diagnoses.

## 3. Results

### 3.1. Cohort Characteristics

**Figure 1:**
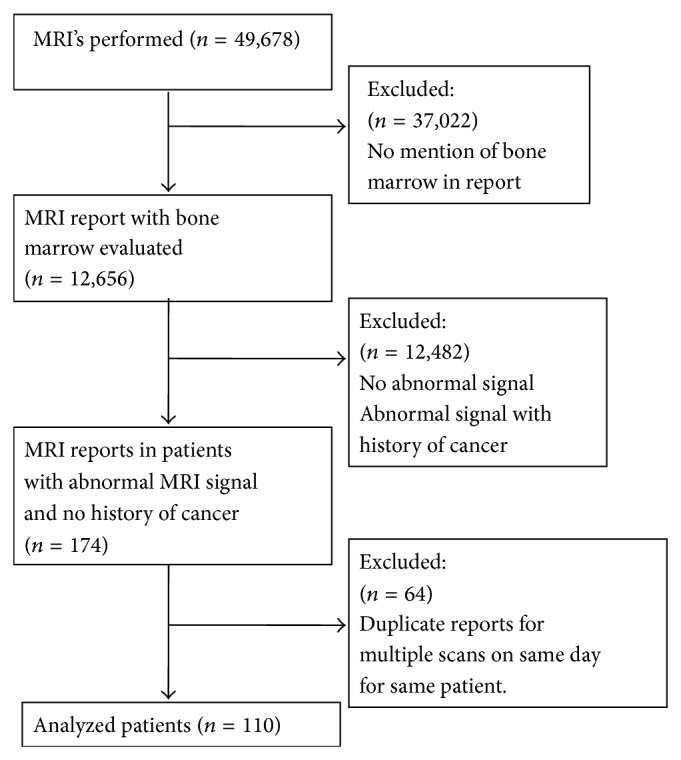
Flow diagram of study.

**Figure 2:**
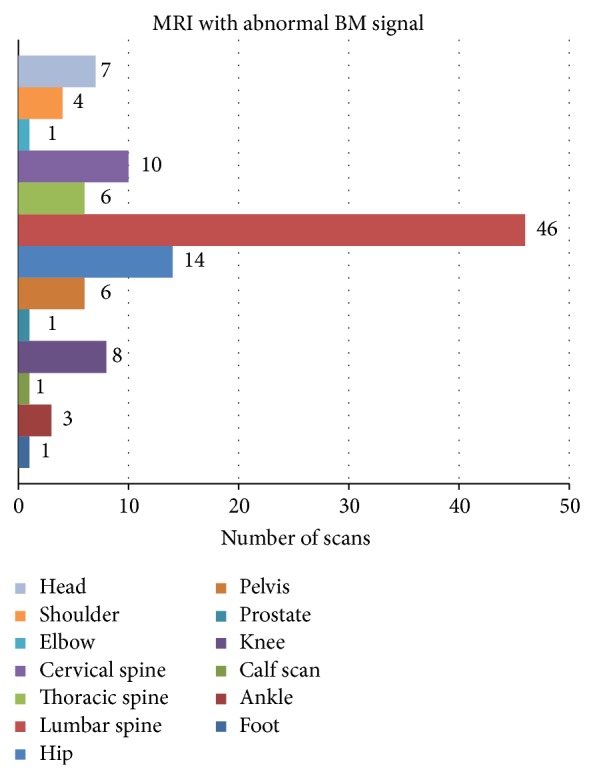
Number of abnormal MRI scans by anatomic location.

Between May 2005 and October 2010, 49,678 MRIs were performed at Tufts Medical Center with 110 patients (pts) meeting search criteria (Figure 1), 47 men and 63 women. Median age was 58.5 years (range, 20–89) with a median follow-up at TMC of 41 months after qualifying MRI (range, 0–103). Thirteen percent had less than 1 month of follow-up. The majority of patients underwent an MRI of the lumbar spine (43%), hip (13%), or knee (7%) (Figure 2), with most of the scans done for musculoskeletal and neurologic complaints (Table 1). Primary care (internal medicine and family practice) was the most common ordering specialty (42%), followed by orthopedics (12%), and neurology (11%). A large portion of the patients were never smokers at the time of the MRI (47%), while only 21% had a hemoglobin of less than 12 mg/dL within 3 months of the scan (Table 1).

### 3.2. Evaluation

**Figure 3:**
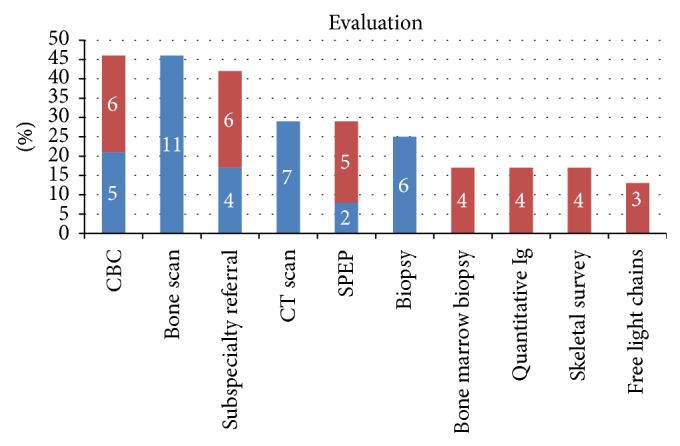
Percentage of evaluated patients (*N* = 110) undergoing each test. Red bars indicate hematology/oncology referral and subsequent testing by hematology/oncology. Numbers on the bars are the actual numbers of patients evaluated with each test.

Twenty four patients (22%) underwent further work-up. Laboratory evaluation included complete blood count (11 pts, 46%), serum protein electrophoresis (7 pts, 29%), quantitative immunoglobulins (4 pts, 17%), and serum free light chains (3 pts, 13%). Further imaging included bone scan (11 pts, 46%), CT scan (7 pts, 24%), skeletal survey (4 pts, 17%), repeat MRI (4 pts, 17%), and DEXA scan (1 pt, 4%). Bone marrow biopsies were done in 17% (4 pts) of those evaluated, while other sites were biopsied in 24% (6 pts) of patients. Subspecialty referrals were made for 16 pts (42%), with 6 (25%) to hematology/oncology, 3 (13%) to neurosurgery, and 1 (4%) to orthopedics (Figure 3). Referral to the surgical specialties prompted follow-up imaging, but no additional laboratory testing. All patients referred to hematology/oncology had abnormal lumbar spine MRIs. In patients who had an abnormal scan but did not undergo any evaluation, 31 (36%) were current or former smokers at the time of the MRI and 23 (20%) had hemoglobin less than 12 mg/dL within 3 months of the scan.

### 3.3. Definitive Diagnosis

A definitive diagnosis was assigned in 11/24 (46%) cases that were evaluated and in none of those that did not undergo work-up. Nonmalignant diagnoses included hemangiomas (3/24), osteoporosis (1/24), and bone bruising (1/24). Malignant diagnoses were identified in 6/24 (25%) patients with 3 patients having multiple myeloma (MM), one mucosa-associated lymphoid tissue (MALT) lymphoma, one metastatic non-small cell lung cancer, and one metastatic adenocarcinoma. One patient who did not undergo any evaluation was later diagnosed with breast cancer at 24 months post scan, resulting in a 7/110 (6%) incidence of malignancy among patients with abnormal bone marrow signal reported on MRI.

## 4. Discussion

Increased demand for hematopoiesis prompts reconversion from fatty marrow to cellularly active red marrow. The pattern occurs opposite to the initial conversion beginning in the pelvis and proceeding peripherally to the long bones [14] and is evident noninvasively on MRI scans. As the skeleton is imaged on scans of many anatomical sites, this incidental finding may be the initial sign of an underlying malignancy in an otherwise asymptomatic patient undergoing imaging for another complaint.

This is the first study to evaluate the incidence and evaluation of abnormal bone marrow signals seen incidentally on MRI. Other studies have examined more global incidental findings on MRIs of specific anatomic areas. High rates of incidental findings have been found on brain MRIs including 0.7–1.6% benign and malignant tumors [15, 16]. Similarly, extraintestinal findings were seen in 25–57% of patients on abdominal MRIs with 0.7% resulting in a cancer diagnosis [13, 17] and clinically significant incidental findings were seen on 10–34% of cardiac MRIs, with malignant diagnoses found in 0.4–4.8% including lung cancers, lymphoma, and thyroid cancer [12, 18, 19]. Furthermore, 10–32% of whole body MRIs had clinically relevant abnormalities requiring work-up with 5.9% identifying a malignancy [10, 20]. Interestingly, abnormal bone marrow signal was not mentioned. In our study, the overall rate of bone marrow signal abnormalities in all patients undergoing MRI was low, but six percent of patients meeting our criteria were subsequently diagnosed with a malignancy.

The lumbar spine was the most commonly imaged site in which an abnormal bone marrow signal was noted. This is likely due to the expected pattern of reconversion starting centrally. Our rates were similar to a study of lumbar spine MRIs which reported that 3.2% of patients were diagnosed with a pathologically confirmed incidental malignancy, with four patients having bladder cell transitional carcinoma, two colorectal cancers, and one prostate cancer extending to the bladder [9]. However, our study had a more even distribution of hematologic malignancies and solid tumors.

Differentiation between malignancies by imaging can be difficult. Hypercellular marrows such as those caused by myeloproliferative diseases and leukemias can range from patchy heterogeneity to diffuse replacement, while cellular depletion states such as aplastic anemia show increased signal intensity on T1-weighted images [21]. Delorme et al. have shown that MM can have focal lesions, homogeneous diffuse infiltration, mixed diffuse and focal infiltration, a variegated salt-and-pepper pattern with scattered fatty islands, or a normal pattern on MRI [22–24]. Marrow infiltration by lymphoma or metastatic solid tumors replaces the fatty marrow with malignant cells and is evidenced on imaging by focal decreases in T1-weighted series. Scans of patients with nonmalignant hematologic diagnoses such as thalassemia and sickle cell disease often show diffuse symmetric reconversion and changes in marrow signal due to iron deposition [11].

Referral to neurosurgery and orthopedics primarily involved evaluation of imaging as opposed to ordering of additional testing. Hematology/oncology referral prompted laboratory and imaging evaluation predominantly for the purposes of identifying MM. As three of the six patients with identified malignant diagnoses had MM, such work-up may not be unreasonable. Three patients were diagnosed with lymphoma and metastatic solid tumors and evaluation of imaging for the patterns described above may help to determine the location to biopsy for greatest diagnostic yield.

Hemangiomas were the most common nonmalignant final diagnosis in our study. Park et al. evaluated the frequency of incidental findings on lumbar spine MRIs and found hemangiomas accounted for 1.5% of the cases [7]. While we found a higher percentage in those undergoing evaluation, the number of hemangiomas overall was similar to the frequency at autopsy [25]. These thin-walled blood vessels form dilated vasculature causing a distinctive mottling of the vertebral bodies and increased signal on both T1- and T2-weighted images [7, 26].

Hip and knee imaging accounted for 20% of the scans with abnormal bone marrow signals. Several studies have assessed the association between pain and bone marrow lesions (BML) over time. These studies found that the size of the BML correlates with pain, though the evidence is stronger for hip than knee. In addition, the lesions changed over time corresponding to pain level and need for surgery [4, 27]. In our study, the majority of scans were done as an evaluation for pain, but there was no mention of following the lesions by the ordering physician.

Other states of reduced oxygen carrying capacity and hypoxia can prompt an increase in marrow reconversion. Examples of such states include high altitude settings, obesity, smoking [5], other pulmonary diseases, and athletes participating in large oxygen debt sports such as long distance running [28]. In addition, metabolism of fatty marrow for energy production can occur in states of extreme cachexia such as human immunodeficiency virus (HIV) infection, chronic renal disease, and anorexia [21]. Questions about new activities, weight changes, or other health abnormalities may point to the etiology if clinical suspicion of malignancy is low.

Several characteristics of the signal can help to differentiate between malignant and nonmalignant reconversion. Malignant etiologies often cause asymmetric reconversion while nonmalignant reconversion is more likely to be bilateral and symmetric. In addition, neoplastic marrow enhances more with gadolinium administration and the signal intensity on short TI inversion recovery (STIR) imaging is greater than the signal intensity of muscle. Furthermore, metastatic lesions tend to localize in the red marrow due to increased blood supply [14]. In our study, we primarily focused on the radiologist's reports and therefore were unable to evaluate the signal characteristics. Discussion with the radiologist may narrow necessity of further testing based on radiographic criteria.

Limitations of this study include follow-up limited to visits at TMC. As TMC is a tertiary care center, several patients were sent only for the MRI with no other encounters within our system or only saw specialists and had their primary care and possible evaluation elsewhere. Inclusion of all patients may have biased the results towards infrequent work-up and diagnosis. If those patients with less than one month of follow-up are excluded, there is a 7/95 (7.4%) incidence of malignancy in our series. Furthermore, it was not possible to delineate what prompted further evaluation and, therefore, a systemic issue we did not collect may have had diagnostic significance. As this was a retrospective study abstracting from the EMR, it is unclear if these findings and possible work-up were discussed with the patient and not documented.

Finally, we attempted to account for other nonmalignant etiologies by evaluating the relationship between hemoglobin and smoking at the time of the scan. However, other nonmalignant etiologies were difficult to determine from the available records. Further studies may help to elucidate the appropriate order of work-up.

In summary, incidentally noted abnormal or heterogeneous bone marrow signal on MRI was not inconsequential. Of those patients who met our study criteria, 6% were diagnosed with a malignancy. We conclude that abnormal bone marrow findings on MRI should not be ignored and initial evaluation by primary care physicians could include identification of cancer risk and possibly laboratory evaluation for multiple myeloma.

## Figures and Tables

**Table 1:** Cohort characteristics.

	All patients *n* = 110, (%)	Evaluated *n* = 24, (%)	Not evaluated *n* = 86, (%)
Age, median (range), and years	58.5 (20–89)	58.5 (24–83)	59 (20–89)
Male gender	47 (43)	8 (33)	39 (45)
Follow-up, median, and months	41 (0–103)	42.7 (0.3–96)	39.6 (0–103)
MRI location			
Lumbar spine	46 (42)	12 (50)	34 (40)
Hip	14 (13)	2 (8)	12 (14)
Cervical spine	10 (9)	3 (13)	7 (8)
Knee	8 (7)	1 (4)	7 (8)
Reason for scan			
Musculoskeletal complaint	67 (61)	13 (54)	54 (63)
Neurologic complaint	26 (24)	7 (29)	19 (22)
Infection	4 (4)	0 (0)	4 (5)
Prior abnormal imaging	3 (3)	1 (4)	2 (2)
Other	9 (8)	3 (13)	6 (8)
Ordering specialty			
Internal medicine	42 (38)	8 (33)	34 (40)
Orthopedics	13 (12)	2 (8)	11 (13)
Neurology	12 (11)	3 (13)	9 (10)
Other	43 (39)	11 (46)	32 (37)
Smoking			
Current smoker at scan	20 (18)	4 (17)	16 (19)
Former smoker at scan	19 (17)	4 (17)	15 (17)
Never smoker	52 (47)	15 (63)	37 (43)
Unknown	19 (17)	1 (4)	18 (21)
Hemoglobin within 3 months of scan			
mgdL	23 (21)	6 (25)	17 (20)
mgdL	46 (42)	11 (46)	35 (41)
Unknown	41 (37)	7 (29)	34 (39)
